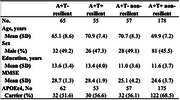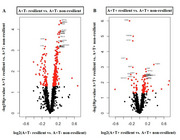# The Underlying Pathophysiology of Resilience to Alzheimer's Disease Pathology: Preliminary Results of a CSF Proteomics Study

**DOI:** 10.1002/alz70856_099706

**Published:** 2025-12-24

**Authors:** Jolien van der Velden, Aurore Delvenne, Stephanie J. B. Vos, Johan Gobom, Betty M. Tijms, Suzanne E. Schindler, Inez H.G.B. Ramakers, Henrik Zetterberg, Pieter Jelle Visser, Willemijn J. Jansen, EMIF‐AD MBD study group

**Affiliations:** ^1^ Alzheimer Center Limburg, School for Mental Health and Neuroscience, Maastricht University, Maastricht, Limburg, Netherlands; ^2^ Alzheimer Center Limburg, School for Mental Health and Neuroscience, Maastricht University, Maastricht, Netherlands; ^3^ Clinical Neurochemistry Laboratory, Sahlgrenska University Hospital, Gothenburg, Gothenburg, Sweden; ^4^ Department of Psychiatry and Neurochemistry, Institute of Neuroscience and Physiology, The Sahlgrenska Academy, University of Gothenburg, Mölndal, Sweden; ^5^ Alzheimer Center Amsterdam, Department of Neurology, Vrije Universiteit Amsterdam, Amsterdam UMC location VUmc, Amsterdam, Netherlands; ^6^ Department of Neurology, Washington University School of Medicine, St. Louis, MO, USA; ^7^ Knight Alzheimer Disease Research Center, Washington University School of Medicine, St. Louis, MO, USA; ^8^ Clinical Neurochemistry Laboratory, Sahlgrenska University Hospital, Gothenburg, Sweden

## Abstract

**Background:**

Up to 25% of individuals with AD pathology (amyloid‐β deposition and tau neurofibrillary tangles) remain cognitively healthy throughout life, showing resilience to AD. The neurobiological mechanisms behind this resilience remain largely unknown, yet understanding this could inform preventive interventions against AD. We aim to investigate the underlying pathophysiology of resilience to AD using CSF proteomics.

**Method:**

355 participants from the EMIF‐AD MBD, Washington University Knight ADRC and Maastricht BB‐ACL studies were included. Resilience was defined as cognitively healthy status with abnormal CSF amyloid‐b_42_ (A+; data‐driven cut‐offs) and (ab)normal phosphorylated tau181 (T+/‐; cohort‐specific cutoffs).) Resilient participants (A+T‐ (*n* = 65); A+T+ (*n* = 55)) were contrasted with persons with MCI or AD dementia with the same pathological profile (A+T‐ (*n* = 57); A+T+ (*n* = 178)). CSF proteomic data were generated using tandem mass tag spectrometry. Protein concentrations were compared between groups using ANOVA adjusted for age, sex and APOEε4 carriership. Proteins measured in at least one‐third of participants across all groups were included, resulting in 1,386 proteins.

**Result:**

Sample characteristics are in Table 1. In A+T‐ resilient participants compared to A+T‐ non‐resilient participants, 221 proteins were significantly upregulated and 75 were significantly downregulated (Figure 1A). Gene ontology (GO) pathway analyses showed that upregulated proteins were mostly associated with nervous system development, ion response, and amyloid fibril formation, while downregulated proteins were mostly related to the innate immune system, hemostasis and proteolysis. In A+T+ resilient participants compared to A+T+ non‐resilient participants, 65 proteins were significantly upregulated and 38 were significantly downregulated (Figure 1B). GO pathway analyses showed that upregulated proteins were mostly associated with oxidative stress and synaptic processes, while downregulated proteins were mostly enriched for the adaptive immune system.

**Conclusion:**

Resilience to AD is associated with unique protein expression profiles and underlying biological pathways, that vary depending on tau pathology status. Resilience in A+T‐ individuals is linked to pathways involved in nervous system development, ion response, and amyloid fibril formation, whereas resilience in A+T+ individuals is associated with oxidative stress regulation and synaptic processes. The distinct protein expression patterns observed suggest that different biological mechanisms may contribute to cognitive resilience, and these mechanisms could change as tau pathology progresses.